# Periostitis—Its Causes and Remedies

**Published:** 1879-08

**Authors:** W. R. Holmes

**Affiliations:** Macon, Ga.


THE
### AMERICAN JOURNAL
OF
### DENTAL SCIENCE.

**Vol. XIII. THIRD SERIES—AUGUST, 1879. No. 4.**

### ARTICLE I

### *Periostitis—Its Causes and Remedies.*

BY DR. W. R. HOLMES, MACON, GA.

Read before the National Dental Association, and Georgia State Dental Association, July 9th, 1879.

Having been appointed at the last meeting of this honorable body, held in Atlanta, last year, to prepare an essay on the subject of Periostitis, to be presented at this meeting, for your consideration, I will now, to the best of my ability, give you such ideas as have occurred to me.

By way of introduction, allow me to state that I feel my inability to lay before you any new or advanced views on the subject, differing, probably, from those held by every member of the society. The subject, however, is an important one, and while I may only give a rehash of old theories, still merely bringing them afresh to your minds, will at least cause a discussion, and mayhaps, here and there among you new ideas and experiences crop out which will be of great value one to tne other.

Periostitis, as its name indicates, means an irritation of the lining membrane, called the periosteum, around the roots of the teeth, located in the alveolus. The Periosteum, the seat of the disease, is a highly vascular membrane, and is subject to considerable degree of inflammation. Perhaps there is no membrane in the human organism, which has more exciting causes to abnormal disturbance, being the lining membrane around the roots of the teeth, and acting, as it were, a cushion between the roots and the inner surface of the alveolar process, thus establishing a close relationship to the teeth, so that whatever causes disturbance in those organs, correspondingly acts upon the membrane. The teeth are constantly in a state of disease, and correspondingly the Periosteum Periostitis, the chief disease to which the membrane is liable, comes probably under the care and observation of the dentist more than any other disease, and none other which requires fewer sense of dognosis or more active and delicate treatment. The disease may exist either in a chronic or active condition. The active originates from direct local irritation. The chronic from systemic influences. The evidence of active periostitis is a dull, heavy, gnawing pain, and is the result of simple vascular excitement—in the ratio of advanced inflammation, the pain increases. The tooth becomes very tender to the touch—in fact becomes longer—so much so, that occlusion of the jaws produces intense pain, and the attention of the sufferer is constantly engaged in keeping the teeth from coming in contact.

If the primary inflammation is not arrested the action goes on until suppuration takes place, and an alveolar abscess the result.

Chronic periostitis originates from many causes.

Mercurial ulitis, a very frequent cause, Scorbutus found very commonly where polk is used exclusively to the neglect of vegetable diet.

Neuralgia reflection frequently causes irritability in the peridental tissue. Rheumatism and gout too often indirectly beget periosteal disturbance. Old age is still another cause, the teeth becoming loose from a degenerating condition of the periostial membrane.

Accumulation of tartar is probably the most common cause where the membrane become excessively irritated. In chronic periostitis the tooth becomes sore and tender and elongated; but the progress is slow, and may exist a long while without very acute pain; the disease is recognized, but is sufficiently bearable, and does not demand active treatment. It most generally though, grows into the active form, and then active remedies are called for; we have therefore, on account of immediate relief being demanded by the sufferer, to deal with this class of periostitis more generally than the chronic. The causes which originate the acute form of periostitis are varied.

The most common probably, originates from a diseased state of the dental nerve. A devitalized nerve will produce acute inflammation, especially should the nerve be confined in the nerve canal and no outlet save that of the foramen at the end of the root for the gas generated from a decomposing nerve to escape through. A blow upon a live tooth causing death of the nerve, brings about this condition, or a filling upon an exposed or nearly exposed nerve, or wedging heroically or using soft rubber between the teeth to separate. Still again the use of ligatures, in the correction of irregularity allowing the ligature to work its way under the gum will cause acute periostitis. Too strong escharotic such as caustic, applied to arrest hemorrhage will bring about this condition. Arsenic applied to an exposed nerve and allowed from an improper stopping of the cavity to drip around the neck of the tooth will cause extreme periosteal disturbance, and frequently absorption of the alveola process, and sometimes entire loss of this membrane.

A crowded state of the teeth, particularly when there is a full denture and the wisdom teeth to come and no room for them, and the wisdom teeth wedging the others forward, causes general periostal trouble, wearing down the teeth, especially found in old people, most frequently from use of tobacco; the enamel and dentine is whetted off until only a thin plate of dentine is left to cover and protect the nerve, causes irritation of the nerve and following inflammation of the periosteum.

These causes just enumerated, are the most common met with in general run of practice. We sometimes find a severe periosteal disturbance, and fail to trace to any of the ordinary causes and are much puzzled to find the origin of the irritation and acute pain. These cases may be termed anomalous.

I will describe a case in practice to illustrate:

A lady of about forty years of age; of full habits, large, robust and healthy, called, complained of soreness in superior right, second molar, and occasional paroxysms of pain darting around the teeth and in the cheek and face; said she had been suffering some time; examined teeth closely, seemed to be perfectly sound, saw no sign of decay. It occurring to me that there might be decay between the teeth, the first and second molars were wedged apart, and sure enough two small decays found. There being no indication of the pulps being dead, the cavities were prepared and filled, the gums painted around with Tinct. of Iodine and Aconite Root, and patient dismissed.

Several days elapsed, and patient returned, still complaining of soreness and occasional paroxysms of pain. Again painted gums with Iodine preparation and had her to come several days, using same treatment Still no relief. Finally decided to drill into pulp chamber of second molars; did so, and found nerve alive and excessive hemorrhage from puncturing nerve; applied arsenious acid, and in due time removed devitalized nerve; treated until all soreness left; when the tooth was filled temporarily, and patient again dismissed. Again she came in, old symptoms, only in the first molar; decided to treat that in same manner as with second molar; did so, and with like result. Patient dismissed; in about two weeks she returned; soreness all gone from first and second molars, but most fearful pains darting about the face; said she would rather have every tooth in her mouth extracted than suffer such agonies. Described her suffering as perfectly excruciating—not continued but at intervals—simply passing her hand over her face, washing her face, holding her head down, touching the tooth with her tongue; in short, any movement of the jaws would produce sudden and darting shocks like a galvanic battery. Examined her mouth again and found the pain seemed to start from the right superior cuspid or eye-tooth, which ap peared to be sore and elongated, and very tender to the touch; especially so to the occlusion of the jaws, indicating extreme periostitis. Decided to try same treatment as pursued in the first and second molars.

Applied arsenious preparation, in due time removed nerve, treated until all soreness was gone and put in a permanent filling, also at same time removed temporary fillings in first and second molars and filled permanently. All pains hav ing disappeared and the mouth of the patient perfectly free from any disturbance, the patient was dismissed and from that time, about six or eight months since to the present she has been entirely free from pain.

Here was an anomalous case of periostitis, such as rarely comes under the observation in ordinary run of practice. This is a case that gives food for thought and discussion! What was the origin of this disturbance? Why those darting pains and extreme paroxysms, like galvanic shocks, made more intense by simply washing the face or moving the jaws or any sudden movement of the head? Why did the periosteal disturbance locate in the first and second molars and the teeth apparently perfectly sound, and when the nerves in them is destroyed and all pain removed it should then locate in the eye tooth; and then why, after that was treated in same manner as the other teeth all the disturbance should entirely disappear? We would like to have an expression of opinion and hope to get more light on so strange a case. Garretson, in his work on Oral Diseases and Surgery, says: "In rare cases there is found to exist an irritability of the dental pulp, which exhausts itself in the formation of isolated granules of semi-bone like characters, which obtain lodgement in some portion of the organ and become in turn the source of great offence to the parts, resulting, indeed, frequently in an odontalgia, than which there are few severer forms. To diagnose this condition, is an exceedingly difficult matter, and it can, perhaps, be best done by exclusion. The teeth in these cases present every appearance of the highest health; no discoloration; no soreness on pressure, and not unfrequently being without the slightest local pain, this manifestation being situated in some distant part, as the ear, eye, the scalp, etc. Whether, however, the pain may be localized or diffused, it is always expressed by the patient as being entirely unbearable, and is commonly, more or less paroxysmal in character, thus being mistaken for idiopathic neuralgia, and frequently so treated."

I quote thus from Garretson. By way of hinting a probable cause for the origin of this case, I will now give a case in practice to show how we are sometimes mistaken in locating periostitis and the cause: A gentleman called, complaining of severe pain in first inferior lower molar—left side—which had been filled by us, probably a year previously; complained of its being sore, when he closed his mouth, in fact, seemed longer than the others when he closed his mouth. We examined and found a large gold filling in the crown. We at first supposed the nerve had died and decided to open into the pulp chamber. The filling being so large, in such perfect condition, we concluded, upon second thought, to apply Iodine and Aconite preparation, and wait for further developments; did so, and asked him to come next day. At the appointed time he came and still complained. In the course of conversation with him, we learned that he had broken off a wooden tooth pick between the first and second molars above the molar complained of, and didn't know whether he had gotten all of it out or not. We examined between the teeth, and sure enough found the tooth pick there and pulled it out. We then applied the Iodine and Aconite preparation a few days, and all trouble ceased. In his case the tooth pick had caused the periosteal membrane to become highly irritated and inflamed; so much so, as to cause the superior molar to become elongated and striking against the lower molar, made him think that this was the offending organ.

To locate the periosteal disturbance and get at the cause, requires care and oftentimes the very closest attention and finest discrimination. The remedies used in periostitis are as varied as the different forms of disease.

All causes of the disease being removed, the remedies are generally of a simple character.

If caused from tartar, remove that carefully and paint around the gums with simple Tincture Iodine and Aconite preparation, and use some carefully prepared mouth wash to restore the gums to their normal condition. If periostitis comes from a dead pulp, remove all traces of the dead nerve and then treat the inflamed membrane on general principles.

Taking it for granted that all causes of inflammation have been removed, a large per cent. of acute periostitis taken in ime may be aborted. In first stage, simply painting the gums with Tinct. Aconite $\frac{1}{4}$ and Tinct. Iodine $\frac{3}{4}$ parts will give relief.

If the form of inflammation be aggravated, provoke free bleeding by scarifying the gums; as soon as the vessels and capillaries have disgorged themselves, paint the parts with odine and Aconite preparation. Place the feet of the patient in very warm water, apply a blister the size of a dime piece just in front of the ear and upon the nape of the neck, about the size of a silver dollar; administer internally twenty-five grains of Bromide of Potassium, having combined with it five drops of the Tinct. Veratrum Veride; this combination may be repeated in four hours if relief is not obtained sooner.

After lancing the gums freely and the use of Tinct. Aconite Root and Iodine preparation, keep cotton saturated with Tinct. Calendula, or if this is not convenient, with the fluid extract of Hamamelis Virginicus combined with equal parts of Phenol-sodique. This treatment, if all causes of irritation have been removed, seldom fails to relieve. It will be well to keep the feet in warm water until the patient grows faint or breaks into perspiration. To those of a plethoric temperament half ounce of Sulphate of Magnesium may be given in a goblet half full of water.

Hydrate of Chloral is often used with efficacy; about ten grains to the dose is a sufficient quantity to be administered and will always be on the safe side.

Leeches are sometimes used to advantage placed on the gums at the root of the offending organ; either in the lower or upper maxillary. This is very objectionable to some patients and cannot always be used. A simple remedy is the application of a roasted raisin over the root; this sometimes is found beneficial.

A valuable refrigerant lotion to the inflamed parts may be applied on a pledget of cotton placed between the gum and cheek, is as follows:

℞.—

| | |
|---|---|
| Plumbi Acitatis, - | 1 drachm, |
| Tinct. Opii, - | ½ ounce, |
| Aquæ, - - | 10 ounces. |
| | Mix. |

Another:

℞.—

| | |
|---|---|
| Potassa Chlorate, - | 2 ounces, |
| Aquæ font., - | 8 ounces, |
| Tinct. Opii, - - | ½ ounce. |
| | Mix. |

Dr. Chase, of St. Louis, Mo., recommends in incipient periostitis, saturated solution of Iodide Potassium, one drop every hour until better. Another of his remedies in mercurious vivus, third decimal trituration. One grain every hour until better. No medication, he says, is more efficacious than this homœpathic remedy as prepared by them.

There are many other good remedies used by different members of the profession, and we trust that each member of the Society will ventilate his particular remedy and mode of treatment, so we can each derive benefit from it in our practice. We are here to exchange ideas and learn from each other.

After years of study and labor, who among us is satisfied with the work of his hands; how many imperfections we see; desire is not satisfied; rest is not found.

There are those mayhaps, who are so self-sufficient as to flatter themselves that their productions are perfect. To such, an appeal would be folly, for "egotism has dammed up the sponge of their aspirations and ignorance holds unlimited sway."

There is probably no one part of the body, the treatment of which requires such an extensive knowledge of the system as the mouth and its attendants.

It is the duty of the Dentist to treat all parts that surround the teeth and are influenced by them, or which they influence.

All should labor zealously for higher attainments. We must labor for proficiency; "to save is better than to destroy." Dental art should be more conservative than it has been. The forceps should rust more in idleness instead of being kept bright with active service.

The preservation, not extraction of a tooth, will then, as it should now, enlist all the energies of the devoted and conservative dentist, who, with the co-operation of the patient, when he has been well trained, will be able to succeed in saving from "the rapacious jaws of the bloody forceps," God's beautiful creation, "those pearls of great price."

---

